# Essential Oils of Lamiaceae Species as Promising Sources of Antimycobacterial Agents: Current Insights, Challenges, and Future Perspectives

**DOI:** 10.3390/molecules31183295

**Published:** 2026-09-17

**Authors:** Ana Clara Aprotosoaie, Mihaela Lipovanu, Adina Catinca Grădinaru, Anca Miron

**Affiliations:** Faculty of Pharmacy “Grigore T. Popa”, University of Medicine and Pharmacy Iasi, 16 Universitatii Street, 700115 Iasi, Romania; ana.aprotosoaie@umfiasi.ro (A.C.A.); mihaela.gafton@umfiasi.ro (M.L.)

**Keywords:** Lamiaceae, essential oils, antimycobacterial activity, *Mycobacterium tuberculosis*, drug resistance

## Abstract

Tuberculosis remains a major global health threat, severely exacerbated by the emergence of multidrug-resistant strains. The bioprospecting of natural matrices for novel, efficacious, and safe antimycobacterial agents represents an important strategy within the drug discovery pipeline. Essential oils (EOs) derived from the Lamiaceae family represent a rich reservoir of bioactive constituents, with well-documented antimicrobial properties and broad therapeutic potential. This review evaluates the current state of research regarding the antimycobacterial activity of Lamiaceae EOs, highlighting their efficacy against *Mycobacterium tuberculosis* resistant phenotypes, mechanism of action, structure-activity relationships, synergistic interactions with conventional antitubercular chemotherapies, and existing translational challenges. EOs from species belonging to genera such as *Thymus*, *Mentha*, *Ocimum*, *Salvia*, *Satureja*, *Tetradenia*, and *Zataria* showed significant antimycobacterial potency, with minimum inhibitory concentrations (MIC) below 100 μg/mL. The phenolic monoterpenoids thymol and carvacrol, alongside the abietane-type diterpene 6,7-dehydroroyleanone, exhibited notable antimycobacterial activity with MIC values of 0.78–12.5 μg/mL. Carvacrol and thymol disrupted the lipid-rich mycobacterial cell wall, increased bacterial membrane permeability, impaired energy metabolism, and induced oxidative stress. These compounds also exerted dual inhibitory effects on biofilm formation and development, interfered with Rv1258c and Rv0194 efflux pumps, and inhibited chorismate mutase activity. Similarly, 6,7-dehydroroyleanone compromised the integrity of the lipophilic mycobacterial cell envelope, suppressed cell wall biosynthesis, blocked ABC-family efflux transporters, and induced intracellular oxidative stress. Lamiaceae EOs exhibit multi-target antimycobacterial efficacy and hold considerable potential as adjuvants in combination antituberculosis regimens. To facilitate the translation of these natural matrices from bench to bedside, future research should focus on robust microbiological and chemical standardization, pharmacological and toxicological evaluation, and the development of advanced nanoformulations of EOs to optimize bioavailability and mitigate host cytotoxicity.

## 1. Introduction

Tuberculosis (TB), a long-standing infectious disease caused by *Mycobacterium tuberculosis*, continues to be a major global health concern. In 2024, TB affected 10.7 million people worldwide, including 1.2 million children, and an estimated 1.23 million people died of TB [1,2,3]. According to the WHO, TB was the leading cause of mortality from a single infectious agent in 2024, and TB is among the top ten causes of death worldwide [2]. The emergence of multidrug-resistant (MDR) and extensively drug-resistant (XDR) isolates is a public health security threat and a significant challenge for the current pharmacotherapy of active tuberculosis. The global burden of multidrug-resistant tuberculosis (MDR-TB) is considerable, with about 390.000 patients developing MDR-TB in 2024 [2]; additionally, it is appreciated that 25% of TB-related deaths are attributed to MDR/XDR infections [4]. MDR-TB is characterized by mycobacterial resistance to the frontline antitubercular agents isoniazid and rifampicin. XDR isolates of *Mycobacterium tuberculosis* exhibit this baseline resistance profile alongside resistance to fluoroquinolones and at least one of the three second-line injectable drugs (amikacin, capreomycin, and kanamycin) [5,6]. The picture of microbial resistance is further complicated by the emergence of newly classified resistant pathogens, namely extremely drug-resistant (XXDR; they are resistant to all first- and second-line drugs) and totally drug-resistant strains (TDR; resistant to all currently available antibiotics, as well as some antibiotics still in the discovery pipeline) [7,8]. The increasing prevalence of *Mycobacterium tuberculosis* drug-resistant phenotypes, together with the adverse effects of long-term antitubercular pharmacotherapy, underscores the need for effective drugs with improved pharmacokinetic profiles and pleiotropic mechanisms of action and molecular targets. Natural products, particularly medicinal plants, remain one of the most valuable sources for drug discovery. Approximately 40% of currently available pharmaceuticals are sourced from plants; four of the first-line antitubercular medicines (ethambutol, isoniazid, pyrazinamide, and rifampicin) were developed from natural products [9].

The Lamiaceae family, commonly known as the mint family, is one of the most significant groups of flowering plants, encompassing over 236 genera and more than 7000 species widely distributed from the Mediterranean basin to tropical and temperate regions. The prominent genera of the mint family include *Mentha*, *Ocimum*, *Origanum*, *Rosmarinus*, *Thymus*, *Salvia*, and *Satureja*, all of which are well-known aromatic herbs largely used for culinary and medicinal purposes. Plants belonging to the family Lamiaceae are among the most important medicinal plants with a significant global economic value for pharmaceutical, food, cosmetic, perfumery, and agrochemical industries. In addition, they play significant roles in chemotaxonomy, biodiversity, and ecology. They produce specialized metabolites such as essential oils (EOs) rich in monoterpenoids and sesquiterpenes, and non-volatile compounds (diterpenes, triterpenes, flavonoids, and other phenolic compounds, including rosmarinic acid). These metabolites exhibit diverse and potent biological activities, including antimicrobial, antioxidant, anti-inflammatory, neuroprotective or chemopreventive effects [10,11]. Owing to their extensive ethnopharmacological validation and rich chemodiversity, Lamiaceae species constitute a valuable reservoir for drug discovery, natural product chemistry, and pharmacological research [12]. A comparative taxonomic screening of traditional medicinal plants used against tuberculosis, based on their antimycobacterial efficacy, ranked the Lamiaceae family among the top three most prominent families, following Asteraceae and Fabaceae [13].

The antimycobacterial efficacy of Lamiaceae EOs has been documented in various studies, typically within the broader context of the antimicrobial properties of EOs or the antitubercular effects of phytocompounds/natural products. However, a systematic and cohesive framework characterizing the antimycobacterial potential of these volatile products remains lacking in the current literature. To address this knowledge gap, the present review provides a synthesis and a critical analysis of existing experimental data in assessing the antimycobacterial activity of Lamiaceae EOs, with particular emphasis on those exhibiting activity against *Mycobacterium tuberculosis* MDR strains. Furthermore, the review addresses the underlying molecular mechanisms of action, the ability of Lamiaceae EOs to counteract microbial resistance, and the key structure-activity relationship descriptors that dictate the antimycobacterial potency. In addition, the synergistic interactions between Lamiaceae EOs and conventional antitubercular therapies, together with their prospects for clinical translation, are also explored. The limitations of current methodologies, including the limited correlation between in vitro and in vivo data and the bioavailability of EOs, are also discussed, and strategic directions for future research are outlined.

## 2. Methodology

The analysis includes the studies published between 2006–2026. The literature search was conducted across major electronic scientific databases, including ScienceDirect, Wiley Online Library, SpringerLink, Taylor &Francis Online, Oxford Academic, SAGE Journals, Frontiers, Thieme, and PubMed, using specific keywords, such as antimycobacterial, antituberculosis, *Mycobacterium tuberculosis* and MDR-*Mycobacterium tuberculosis*, together with terms referring to Lamiaceae species (*Ocimum*, *Origanum*, *Mentha*, *Thymus*, *Salvia*, and *Satureja*), essential oils and major bioactive compounds such as thymol, carvacrol, citronellol, eugenol, menthol, and β-caryophyllene.

## 3. *Mycobacterium tuberculosis* and Microbial Resistance

**Figure 1:**
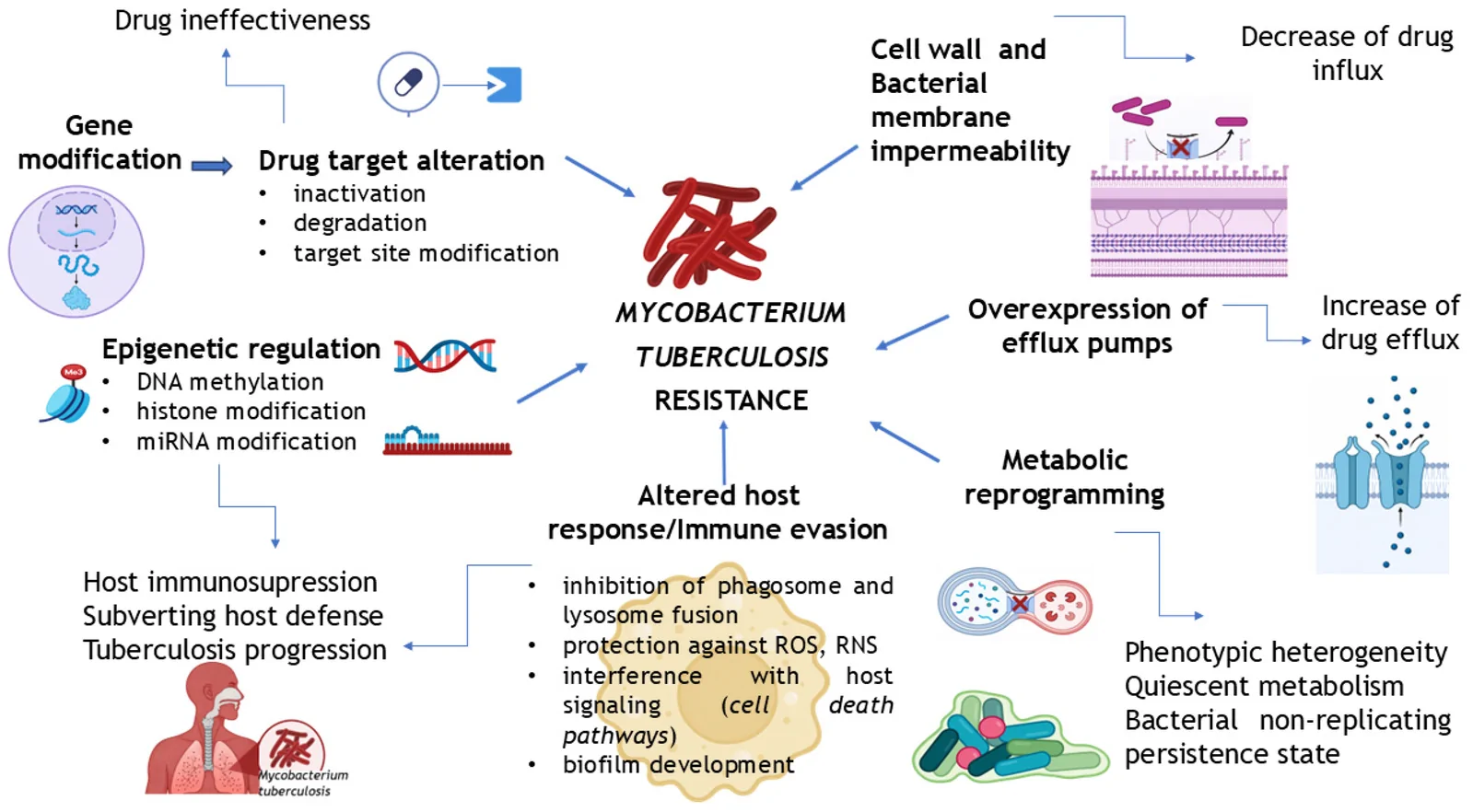
Molecular mechanisms and cellular pathways underlying drug resistance in *Mycobacterium tuberculosis*. Schematic representation of the interconnected genetic, biochemical, and physiological mechanisms contributing to phenotypic and genotypic resistance in *Mycobacterium tuberculosis*. Cell envelope impermeability and efflux pump overexpression restrict drug intracellular penetration and intracellular accumulation. Specific chromosomal mutations disrupt drug-target interactions. Bacterial and host-directed epigenetic regulation can modulate antimicrobial response and impair host-mediated bacterial clearance. Metabolic dormancy and phenotypic tolerance, involving the transition of bacilli to a non-replicating, low-metabolic state, reduce antibiotic efficacy without altering the genomic sequence. Host immunoevasion. Active bacterial interventions disrupt host macrophage defense by phagosome maturation arrest, inhibition of phagosome-lysosome fusion, neutralization of oxidative stress, and modulation of innate signaling and cytotoxicity, promoting phenotypic tolerance and disease progression [20]. The illustration includes graphic elements adapted from BioRender.com.

Mycobacteria are Gram-positive, aerobic, non-motile, and pleiomorphic bacili [14]. These intracellular pathogens have a sophisticated architecture of the cell envelope that highly supports microbial resistance. The mycobacterial cell envelope consists of a cell wall, periplasmic space, and plasma membrane [15]. The mycobacterial cell wall presents a three-layered structure of covalently linked lipids and polysaccharides with unique chemical features. It is extremely rich in complex lipids that represent up to 60% of the bacterial lipid content, providing a highly hydrophobic environment and an efficient barrier to antibiotics and chemotherapeutic agents or components of host immune defense response [14]. A hallmark of the genus *Mycobacterium* is the presence of mycolic acids, unusually 2-alkyl, 3-hydroxy long-chain fatty acids (C60–C90) which are found in the outer layer of the mycobacterial cell wall. They are associated with complex lipids such as trehalose monomycolate and dimycolate, phospholipids, glycopeptidolipids, phthiocerol dimycocerosate, phosphatidylinositol mannosides, lipomannan, lipoarabinomannan, and sulfolipids [15]. The innermost layer of the *Mycobacterium tuberculosis* cell wall is composed of highly cross-linked peptidoglycan (PG) chains, which provide structural integrity and protection in the microenvironment. The PG units are covalently attached to arabinogalactan (AG) strands whose superficial ends are esterified with mycolic acids. The length of the AG chain also affects the shape and hydrophobicity of the mycobacterial membrane. The periplasmic space contains mannose-derived glycophospholipids, whereas the plasma membrane, similar to other bacteria, confers structural integrity [15,16]. Due to this specific architecture of the cell envelope that exhibits low permeability, *Mycobacterium tuberculosis* is inherently resistant to many antibiotics. Apart from the cell wall barrier permeability, drug resistance in *Mycobacterium tuberculosis* occurs by multiple mechanisms, including chromosomal mutations that alter the drug target or the prodrug-activating enzymes, overexpression of efflux pumps, porin downregulation, biofilm formation, compensatory evolution, and target mimicry (Figure 1) [1,7,17,18]. Monotherapy, patient non-adherence, inadequate drug dosages, pharmacokinetic variability, and substandard drug quality can also contribute to the emergence of *Mycobacterium tuberculosis* drug resistance [18,19].

## 4. Antimycobacterial Activity of Lamiaceae Essential Oils

EOs derived from plants of the Lamiaceae family exhibit significant in vitro inhibitory activity against *Mycobacterium tuberculosis*, including both susceptible and MDR strains. Several genera such as *Coleus*, *Mentha*, *Micromeria*, *Ocimum*, *Salvia*, *Satureja*, *Tetradenia*, *Thymus* and *Zataria* have been extensively investigated and showed MIC values ranging from 0.5 to 351.6 μg/mL against standard strains and from 1.465 to 156 μg/mL against MDR clinical isolates (Table 1). The in vitro antimycobacterial activity of several terpenes characteristic of Lamiaceae EOs has also been evaluated. Their MICs typically ranged from 0.78 μg/mL to 2.67 mg/mL on susceptible reference strains and from ≤12.5 μg/mL to 10.68 mg/mL against clinical isolates (Table 2). The studies used the H37Rv (ATCC 27294) and H37Ra (ATCC 25177) as drug-susceptible *Mycobacterium tuberculosis* reference strains; it is worth noting that most of the studies were conducted using H37Rv, a fully virulent international reference strain that requires Biosafety Level 3 (BSL-3) laboratory facilities for handling. H37Ra is an avirulent, attenuated strain that is commonly used in BSL-2 laboratories. There are genetic, phenotypic, and metabolic differences between these two reference strains that promote and support the virulence and survival of H37Rv even in hostile conditions (acidic environments and high-osmolarity conditions). The proteomic analyses showed that the secretion system subunit SecF and ABC-transporter proteins are overexpressed in H37Rv strains, and they play a crucial role in bacterial virulence and host infection [21]. The EOs of *Mentha longifolia*, *Ocimum sanctum*, and *Thymus vulgaris* exhibited the highest antimycobacterial activity against the H37Rv strain with MIC values of 0.8–1.6 μg/mL, 2.931 μg/mL and 0.5–40 μg/mL, respectively. Also, the EOs of *Salvia aratocensis* (MIC = 62.5 μg/mL), *Tetradenia riparia* (MIC = 62.5 μg/mL), *Satureja rechingeri* (MIC = 78 μg/mL), and *Zataria multiflora* (MIC = 78 μg/mL) showed a remarkable antimycobacterial activity against the H37Rv strain (Table 1). Some authors reported that EOs and plant extracts with MIC values ≤ 200 μg/mL exhibit good antimycobacterial activity. EOs or extracts whose MIC values are below 100 μg/mL are considered highly active, whereas those with MIC values from 100 to 500 μg/mL show moderate activity. MIC values between 1000 and 2000 μg/mL indicate poor activity against *Mycobacterium tuberculosis* [22,23]. In this regard, all the aforementioned EOs can be considered highly active against *Mycobacterium tuberculosis*. Furthermore, pure compounds with MIC values ≤ 64 µg/mL are considered promising candidates for antimycobacterial drug development [24]. A threshold MIC value of ≤10 μg/mL is indicative of high antimycobacterial potency [25]. Among the terpenes of Lamiaceae EOs, thymol, carvacrol, β-citronellol, estragole, eugenol, 6,7-dehydroroyleanone, linalool, and menthol exerted good activity against the H37Rv strain, with MIC values as low as 64 μg/mL. The phenolic monoterpenoids thymol and carvacrol proved to be the most active compounds (MIC = 0.78 and 2.02 μg/mL, respectively). Regarding the activity against clinical isolates, *Ocimum sanctum*, *Tetradenia riparia*, *Salvia aratocensis*, and *Zataria multiflora* EOs exhibited potent efficacy; they completely inhibited both susceptible and MDR strains at concentrations of 1.465–5.862 μg/mL, 31.2–62.5 μg/mL, 49.6–99.2 μg/mL, and 78 μg/mL, respectively (Table 1). The MDR clinical isolates tested were usually resistant to first-line anti-tuberculosis drugs, including isoniazid, rifampicin, pyrazinamide, and ethambutol, as well as second-line agents such as streptomycin, kanamycin, cycloserine, and ciprofloxacin. The antimycobacterial activity of *Tetradenia riparia* EO has also been evaluated against *Mycobacterium tuberculosis* strains of the Beijing genotype, which exhibit high virulence and are associated with the spread of MDR-tuberculosis [26]. Among the terpenes commonly found in Lamiaceae EOs, only a limited number have been tested as pure compounds against *Mycobacterium tuberculosis* MDR strains (Table 2). The monoterpene phenol carvacrol (MIC = 19–76 μg/mL) and 6,7-dehydroroyleanone, a characteristic abietane diterpene of *Tetradenia riparia* EO (MIC ≤ 12.5–32.5 μg/mL), are particularly noteworthy. By conjugating thymol and menthol with ciprofloxacin, an old alternative fluoroquinolone used in tuberculosis therapy, Szulczyk et al. (2023) [27] synthesized hybrid derivatives with enhanced lipophilicity and improved activity against MDR-*Mycobacterium tuberculosis* (MIC = 8–32 μg/mL) (Table 2). The menthol-ciprofloxacin hybrid featuring an oxohexyl linker exhibited superior tuberculostatic activity (MIC = 8 μg/mL) compared with ciprofloxacin alone (MIC = 16 μg/mL), due to increased membrane permeability and sustained binding affinity toward mycobacterial DNA gyrase, the specific molecular target of ciprofloxacin [27].

**Figure 2:**
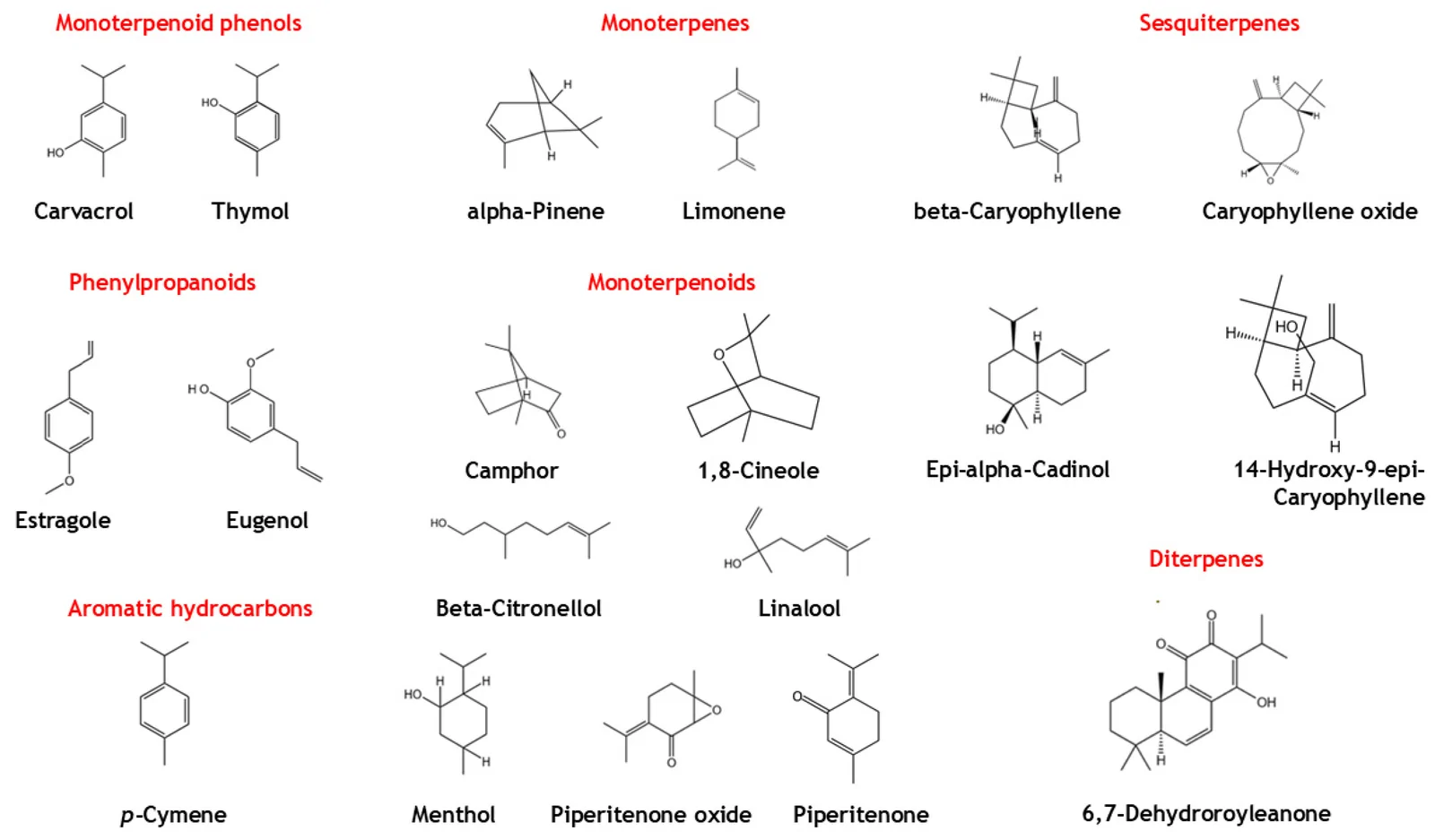
Terpenes in Lamiaceae EOs with antimycobacterial potential.

Figure 2 depicts the chemical structures of the major terpenes found in Lamiaceae EOs that exhibit antimycobacterial activity.

### 4.1. Mechanisms Underlying the Antimycobacterial Activity of EOs

**Figure 3:**
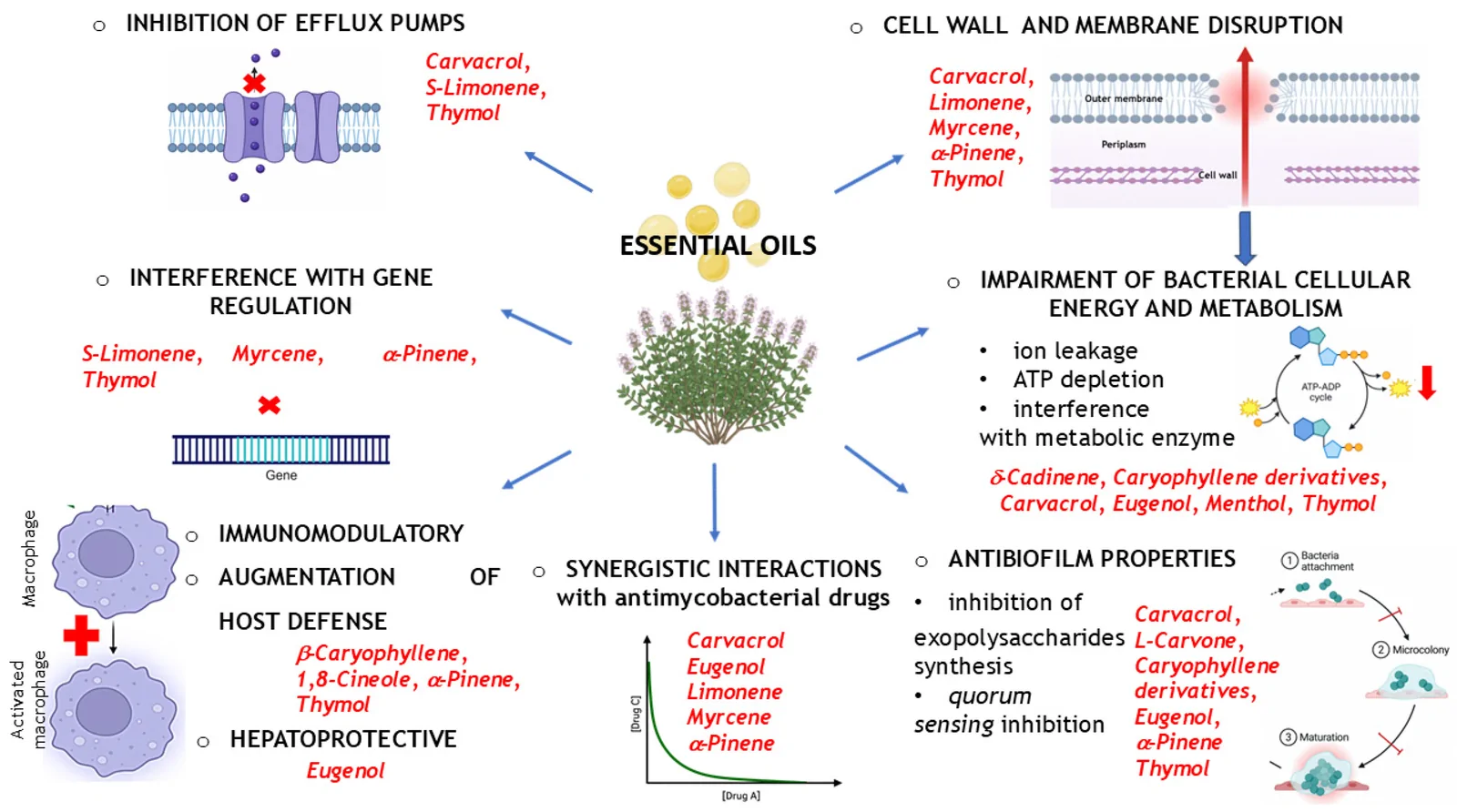
Schematic overview of the antimycobacterial mechanisms of activity of Lamiaceae EOs. The diagram illustrates the multi-target effects of Lamiaceae EOs against *Mycobacterium tuberculosis* cellular integrity, metabolic pathways, genetic regulation, and virulence factors, as well as host immunomodulation and interaction with antimycobacterial drugs. Membrane and cell wall disruption: Lipophilic molecules of EOs permeabilize the mycolic acid layer and cell membrane, and depolarize transmembrane potential, leading to leakage of critical intracellular constituents. Metabolic inhibition: Selective downregulation of ATP synthase complexes impairs cellular respiration, while concurrent induction of intracellular reactive oxygen species triggers oxidative damage to DNA and enzymatic machinery. Inhibition of efflux pumps prevents drug extrusion and restores bacterial susceptibility to antimycobacterial agents. Transcriptional and gene regulation: Volatile compounds penetrate the cytoplasm and downregulate critical genes involved in cell wall synthesis, metabolic homeostasis, and stress response pathways. Antibiofilm activity: EOs penetrate the extracellular polymeric matrix, inhibiting the synthesis of matrix components and signaling networks (*quorum sensing*) required for biofilm maturation and persistence. Host response augmentation: EOs modulate macrophage activity, enhancing the intracellular clearance of mycobacteria. Concurrently, EOs exert hepatoprotective effects by upregulating endogenous antioxidant enzymes and mitigating antitubercular drug-induced hepatic necrosis and inflammation. Interaction with antitubercular drugs: Permeabilization of the mycobacterial cell envelope by EOs and suppression of efflux pumps enhance the intracellular influx of first-line antitubercular drugs (e.g., isoniazid, rifampicin), resulting in synergistic or additive bactericidal effects and potentially reducing the risk for drug resistance. The illustration includes graphic elements adapted from BioRender.com.

Owing to their complex chemical composition, EOs exert multifaceted antimicrobial effects. The hydrophobicity of EOs enables them to permeate the mycobacterial membrane and mycolic acid-rich cell wall more easily, thereby affecting both the structural and functional integrity of *Mycobacterium tuberculosis* cells [22,24,48] (Figure 3).

Terpenoids, mainly phenols and aldehydes, destabilize the lipid-rich cell wall, a critical feature responsible for the intrinsic resistance of mycobacteria to many antitubercular chemotherapeutics. They increase membrane permeability, cause leakage of cytoplasmic content, impair energy metabolism and the function of efflux pumps, and interfere with the biosynthesis of genetic material [6,48,49]. Carvacrol, the major phenol of oregano EOs, induces morphological changes in mycobacterial cells and affects the lipid patterns of cell wall synthesis [39,50]. Significant changes in mycobacterial cell morphology have also been observed following exposure to α-pinene (*Rosmarinus officinalis*, *Salvia officinalis*, *Mentha* sp.), myrcene (*Melissa officinalis*, *Rosmarinus officinalis*, *Lavandula angustifolia*), and limonene (*Mentha* sp., *Dracocephalum* sp., *Agastache* sp.). Exposure of bacteria to these monoterpenes causes cell filamentation, an abnormal growth process in which mycobacterial elongation continues without cell division. This morphological change is triggered by various stress conditions, including oxidative stress, nutrient depletion, DNA damage, and drug exposure [51].

EOs also impair vital metabolic processes, such as ATP synthesis and enzyme activity, leading to cellular energy depletion and inhibition of mycobacterial growth [48].

In silico studies revealed that several terpenes found in Lamiaceae EOs, such as carvacrol, thymol, caryophyllene oxide (*Hyptis stricta*, *Mentha* sp., *Salvia* sp.), germacrene D (*Salvia sclarea*, *Scutellaria volubilis*, *Craniotome furcata*, *Phlomis samia*), cadinene derivatives (*Clinopodium vulgare*, *Melissa officinalis*), R-limonene (*Agastache* sp., *Mentha* sp., *Rosmarinus officinalis*), and α-terpinyl acetate (*Thymus pulegioides*, *Salvia* sp.), can interact with some mycobacterial enzymes that play essential roles in cell envelope integrity, bacterial metabolism and survival, virulence, and microbial resistance (Table 3). Carvacrol, caryophyllene oxide, and cadinene derivatives exhibit strong enzyme-binding affinities, resulting in effective inhibition of enzymatic activity.

Efflux pumps play important roles in cellular homeostasis through the transport of foreign substances and endogenous metabolic waste out of cells [55]. *Mycobacterium tuberculosis* possesses a large number of putative drug efflux pumps that mainly belong to the Major Facilitator Superfamily (MFS), ATP-binding cassette (ABC), and Small Multidrug Resistance (SMR) families. These efflux systems contribute to both innate and acquired drug resistance. Moreover, the efflux-based mechanisms are involved in mycobacterial pathogenicity, virulence, biofilm formation, and the development of early macrophage-induced drug tolerance [55,56,57].

Active drug efflux serves as the primary mechanism driving phenotypic resistance in many clinically resistant *Mycobacterium tuberculosis* isolates [5]. Terpenes present in Lamiaceae EOs inhibit certain mycobacterial efflux pumps and may therefore limit the development of drug resistance.

Thymol effectively targets two key multidrug efflux pumps, Rv1258c (MFS family) and Rv0194 (ABC family); it displays superior activity compared to verapamil, a classical inhibitor of ABC transporters in *Mycobacterium smegmatis*, a surrogate model for *M. tuberculosis*. Both efflux pump proteins contribute to the development of resistance to various chemotherapeutic agents (rifampicin, isoniazid, aminoglycosides, ethambutol, pyrazinamide, and some second-line fluoroquinolones). They are also involved in biofilm formation under hypoxic stress [55,58]. Carvacrol showed efflux pump inhibitory activity in H37Rv comparable to that of verapamil, which was used as the positive control [39].

*Mycobacterium tuberculosis*, as well as other nontuberculous mycobacteria, can form biofilms both in vitro and in vivo. These biofilms are involved in antimicrobial resistance, bacterial virulence, host immune evasion, and infection persistence [59,60]. Bacteria residing within biofilms exhibit 700 times greater resistance to antimicrobial agents than their free-living planktonic cells [61].

Mycobacterial biofilms are dynamic multicellular structures that include bacteria embedded in an extracellular matrix comprising polysaccharides, proteins, DNA, and free mycolic acids. Biofilm-associated bacterial populations represent a critical therapeutic challenge in clinical settings. They exhibit a superior tolerance to conventional drugs and host immune clearance compared to their planktonic counterparts. Biofilm-associated mycobacteria are capable of long-term host colonization, causing latent and chronic infections and increasing the risk of infection recurrence. Beyond functioning as a protective barrier and a nutrient reservoir, the biofilm matrix facilitates critical intercellular communication among resident bacteria. This signaling mechanism, known as *quorum sensing* (QS), is highly associated with and actively regulates the process of biofilm formation [62]. Terpenes of Lamiaceae EOs can disrupt biofilm architecture, inhibit bacterial proliferation, and interfere with QS and adhesion processes [62]. Anti-QS mechanisms include sequestration of QS-mediated molecules, dysregulation of QS gene expression, and inhibition of autoinducer release [63]. Thymol and carvacrol (*Thymus vulgaris*, *Origanum vulgare*) impair the structural integrity and survival of mycobacterial biofilm, interfering with the synthesis of exopolysaccharides. Both compounds also act as QS inhibitors [58,64]. Modelling approaches have shown that, at concentrations below MICs, thymol and carvacrol from *Thymus vulgaris*, as well as L-carvone from *Mentha spicata*, act as bifunctional molecules that reduce acyl homoserine lactone (AHL) synthesis via LuxI-type proteins in many Gram-negative bacteria [65,66]. AHLs are important QS signaling molecules that monitor the bacterial population density and biofilm development, and coordinate QS-related gene expression [67]. LuxI-type proteins constitute a complex regulatory system for AHL-related QS, biofilm production and motility, and virulence factor production in Gram-negative bacteria [68]. Bioinformatic studies have identified LuxR-like transcriptional regulators in mycobacteria [1]. Plant-derived phenylpropenes, specifically eugenol (*Ocimum sanctum*, *O. gratissimum*), can also directly interact with LuxI-type proteins, down-regulating QS signaling molecules in Gram-negative bacterial biofilms. Additionally, eugenol significantly suppresses QS-controlled gene expression and disrupts the biofilm architecture of multi-drug resistant clinical isolates of Gram-negative bacteria by inhibiting extracellular polysaccharide synthesis [65]. Furthermore, pinene derivatives, limonene (*Rosmarinus officinalis*, *Mentha* sp.), and several sesquiterpenes including germacrene D, caryophyllene oxide, and *trans*-caryophyllene have also demonstrated mycobacterial biofilm-reducing properties [54]. Depending on the concentration, chemical composition and microenvironment, EOs can trigger oxidative stress, thereby disrupting cellular redox homeostasis and damaging essential bacterial macromolecules [48]. Compounds such as 1,8-cineole (*Rosmarinus officinalis*, *Mentha* sp., *Lavandula angustifolia*) and thymol have demonstrated immunomodulatory effects, potentially enhancing the host macrophage response to the infection [69]. The essential oils from different parts of *Premna odorata*, containing α-pinene, *trans*-caryophyllene and β-phellandrene as major compounds, showed immunomodulatory effects in murine models infected with *Mycobacterium tuberculosis*. Oral pretreatment with these EOs (300 μL/day, 15 days) enhanced host immune response and antioxidant defense mechanisms via modulation of Toll-like receptor 4 (TLR-4) and nuclear factor kappa light chain enhancer of activated B cells (NF-κB) signaling pathways in TB-infected mice [70]. In addition, the anti-inflammatory and antioxidant effects may alleviate TB-associated lung injury or antimycobacterial drug toxicity and enhance survival outcomes. Eugenol (6 and 12 mg/kg body weight) protected against the hepatotoxicity and nephrotoxicity induced by isoniazid and rifampicin in rats by mitigating oxidative stress [71].

The key structural descriptors for antimycobacterial efficacy of terpenes include the number of conjugated carbons, the number of phenolic and hydroxyl groups, and the number of hydrogen bond acceptor atoms. The molecule’s lipophilicity, the electronic characteristics of the phenolic group, and the position of the hydroxyl group relative to the larger aliphatic chain significantly influence antimycobacterial activity [38,45]. Aromatic compounds and oxygenated monoterpenes are more effective than their corresponding carbonyl compounds [45]. Diterpene quinone derivatives, such as 6,7-dehydroroyleanone (*Tetradenia reparia*, *Plectranthus* sp., *Salvia moorcraftiana*), are also prominent antimycobacterial agents. The presence of a para-benzoquinone ring may contribute to the antimycobacterial potency of these compounds [72].

### 4.2. Interactions with Antimycobacterial Drugs

Microbial resistance is a critical health issue, and the rapid spread of MDR strains requires the development of active novel therapeutic strategies in order to mitigate their occurrence. Due to their potent antimicrobial activity and pleiotropic mechanisms of action, EOs are attractive candidates in combinatorial strategies to overcome microbial resistance. These approaches involve synergistic interactions between EOs/volatile constituents and antibiotics/antimicrobial chemotherapeutic agents. Numerous EOs, including those from several Lamiaceae species such as *Thymus* sp., *Origanum vulgare*, *Mentha piperita*, *Rosmarinus officinalis*, *Lavandula angustifolia*, and *Zataria multiflora*, as well as their isolated constituents, have demonstrated promising synergistic effects when combined with conventional antibiotics against both susceptible and MDR strains [73,74].

For instance, carvacrol-rich EOs (*Thymus maroccanus*, *T. broussinetii*) acted synergistically with ciprofloxacin against *Pseudomonas aeruginosa* clinical isolates. Similarly, the combination of thymol and ciprofloxacin showed synergistic activity against *P. aeruginosa* ATCC 28853, reducing the antibiotic MIC by four- to eight-fold [75]. Combinatorial strategies against *Mycobacterium tuberculosis* are less explored than those reported for other bacteria. Moreover, studies involving Lamiaceae EOs are currently limited to isolated compounds evaluated in combination with standard antimycobacterial agents (Table 4). Subinhibitory concentrations of α-pinene (*Rosmarinus officinalis*, *Satureja montana*), (R)-limonene (*Dracocephalum kotschyi*, *Agastache* sp.), (S)-limonene (*Mentha* sp., *Agastache* sp.), and myrcene (*Ocimum basilicum*, *Thymus vulgaris*) enhanced the activity of ethambutol and rifampicin against the avirulent *Mycobacterium tuberculosis* H37Ra strain. The MIC values of rifampicin were reduced by at least two-fold in the presence of (R)-limonene against *Mycobacterium tuberculosis* MDR isolates and reference strains [45]. Combinations of carvacrol or eugenol with rifampicin exerted synergistic activity against MDR strains. In addition, eugenol synergistically enhanced the activity of isoniazid and pyrazinamide, other frontline antitubercular drugs. The increased activity of antimycobacterial agents in combination with terpenes may be correlated with enhanced antibiotic influx and retention inside the bacterial cells. EO constituents have membranotropic properties that can disrupt the mycobacterial cell envelope and cause membrane permeabilization, enabling increased antibiotic penetration into bacterial cells. In addition, EOs can inhibit multidrug efflux pumps and microbial enzymes, and alter QS. Rifampicin showed positive interactions with volatile terpenes more frequently than isoniazid and ethambutol [45]. While ethambutol and isoniazid target the mycobacterial cell wall through inhibition of arabinogalactan and mycolic acid biosynthesis, respectively, rifampicin exerts its bactericidal effects at the transcriptional level by inhibiting bacterial RNA polymerase [76]. The concurrent activity of terpenes and rifampicin on distinct molecular targets enhances the potential and magnitude of their synergistic interaction. In addition, in the case of rifampicin, both the inhibition of *Mycobacterium tuberculosis* growth and the suppression of microbial resistance are strongly influenced by the area under the concentration-time curve (AUC)/MIC ratio [77]. The membrane permeabilizing effects of volatile terpenes may enhance the intracellular accumulation of rifampicin, thereby reducing the selection of resistant strains.

Further research is required to characterize the interactions between EOs, their isolated constituents and antibiotics against *Mycobacterium tuberculosis*, both in vitro and in clinical settings.

## 5. Limitations and Perspectives

A critical evaluation of the literature data on the antimycobacterial activity of Lamiaceae EOs reveals several limitations of the current research studies. These limitations include:

**Methodological incongruities and lack of standardized protocols**. Differences in in vitro microbiological assays (MABA, REMA, and BD BACTEC MGIT), strain-specific susceptibility profiles, incubation time, EO solubilization protocol, and EO stability during the assay period are some of the methodological aspects that may explain the discrepancies in MIC values reported for the same compounds against *Mycobacterium tuberculosis* strains. Specifically, Andrade-Ochoa et al. (2015) established an MIC of 2.02 μg/mL for carvacrol against the H37Rv strain using the MABA technique [38]. In contrast, Nakamura de Vasconcelos et al. (2018) reported a substantially higher MIC of 76 μg/mL using the REMA method [39]. The MABA assay is considered to provide more reliable sensitivity than the REMA technique due to its commercially available reagent formulation, which reduces interferences from redox reactions and culture medium.

**Chemical profiles of EOs and their intraspecific variation**. The chemical composition of EOs varies depending on geographic area, pedoclimatic conditions, altitude, harvesting stage, plant material processing, and extraction techniques. Furthermore, the presence of chemical polymorphism within natural populations introduces an additional dimension of intraspecific variation. For instance, *Thymus vulgaris* exhibits notable chemical polymorphism, presenting distinct phenolic (e.g., thymol and carvacrol) and non-phenolic (e.g., linalool, geraniol) chemotypes, the former demonstrating significantly greater antimycobacterial activity. Characterization of the chemical profile of EOs is imperative for ensuring the reproducibility of bioassays, phytochemical standardization, and establishment of structure-activity relationships (SARs) relevant to their antimycobacterial efficacy.

**Matrix effects of EOs versus isolated constituents**. EOs are complex, lipophilic matrices whose bioactivity arises from a network of multitarget pharmacodynamic effects, molecular interactions, and pharmacokinetic modulation. Consequently, the bioactivity of whole EOs often differs from that of their isolated constituents. Trace constituents dynamically modulate the biological properties (membrane diffusion and permeabilization) and physicochemical behavior (solubilization and vapor pressure) of major compounds. The assessment and interpretation of EO bioactivity require a holistic framework to integrate the dynamic and complex interactions inherent in their phytochemical matrices.

**Limited evaluation of synergistic interactions between Lamiceae EOs and conventional antimycobacterials as an adjuvant approach to mitigate antimicrobial resistance.**

**Translational gaps and in vivo antimycobacterial activity**. Translating the antimycobacterial efficacy of EOs from in vitro screening to robust preclinical models represents a major methodological and translational bottleneck in this research area. There is a scarcity of in vivo investigations using murine or guinea pig models of tuberculosis to evaluate EOs or their isolated compounds (carvacrol, thymol) that demonstrated high in vitro efficacy against *Mycobacterium tuberculosis*. Moreover, the cytotoxicity of these agents toward host target cells (lung macrophages) remains poorly characterized. To achieve successful clinical translation, an integrated assessment of pharmacokinetic and pharmacotoxicological profiles, systemic bioavailability, and in vivo antimycobacterial potency is essential.

Based on these considerations, future research should focus on establishing standardized microbiological protocols for assessing the antimycobacterial activity of EOs, while also developing nanostructured delivery systems (nanoemulsions, solid lipid nanoparticles, and liposomal formulations) for EO encapsulation. These advanced systems aim to address current EO formulation challenges, improve pharmacokinetic profile and bioavailability, and facilitate site-specific delivery to target tissues, while minimizing cytotoxicity. In addition, SAR modeling represents a valuable approach for exploiting key terpenes of Lamiaceae EOs as scaffolds for chemical derivatization, thereby enabling the development of antimycobacterial derivatives characterized by enhanced bioactivity, superior metabolic stability, and a favorable cytotoxicity profile.

## 6. Conclusions

Tuberculosis remains a formidable global public health threat exacerbated by the emergence of MDR phenotypes and the significant adverse effects associated with conventional antitubercular regimens. The classical “one disease, one target, one drug” paradigm of drug discovery is increasingly inadequate in the face of the complex evolutionary pressure associated with microbial resistance, necessitating a transition toward multi-target approaches to overcome the therapeutic recalcitrance of *Mycobacterium tuberculosis* MDR phenotypes. Exploitation of plant-derived products represents a promising strategy for the development of novel antimycobacterial therapeutics. Historically, natural products have played a key role in drug discovery and the advancement of pharmacotherapy, representing a rich reservoir of compounds with exceptional chemical diversity and intrinsic bioactivity. This significance is underscored by the fact that approximately 75% of clinically approved anti-infective drugs originate from botanical or natural scaffolds [79,80]. Among natural products, EOs emerge as viable candidates that fulfill the stringent criteria of this novel drug discovery framework. Their multicomponent nature allows them to modulate distinct biological targets simultaneously, offering a holistic therapeutic profile with a reduced propensity for bacterial phenotypic resistance. As one of the largest families of flowering plants, the Lamiaceae family is recognized for the biosynthesis of highly bioactive EOs. Characterized primarily by their antimicrobial and antioxidant properties, these metabolites underpin the family’s medicinal importance, economic value, and ecological significance. Numerous in vitro studies have demonstrated the antimycobacterial potential of several Lamiaceae EOs, including their activity against MDR isolates of *Mycobacterium tuberculosis*. However, a critical evaluation of the available evidence reveals several methodological constraints, substantial chemical variability and important translational gaps that collectively impede their progression toward viable clinical therapeutics. Future research in this field should prioritize the integration of rigorous standardization protocols with advanced drug delivery systems, together with pharmacological and toxicological evaluation, to validate both therapeutic efficacy and safety of the most promising EO constituents and formulations.

## Figures and Tables

**Table 1:** Antimycobacterial activity of Lamiaceae EOs.

EO	Main Compounds	MIC (μg/mL)			Experimental	Potential *	Ref.
		H37Ra	H37Rv	Clinical Isolates			
*Hyptis mutabilis* syn. *Cantinoua mutabilis*, topical bushmint (Columbia)	fenchone (17.1%), 1,8-cineole (12.6%), β-caryophyllene (10.9%), bicyclogermacrene (8.7%)	-	125 ± 0.01	-	Colorimetric macrodilution	Good	[28]
*Mentha longifolia*, horse mint (India)	piperitenone oxide (45.9%), piperitone (17.5%), β-caryophyllene (10.2%), germacrene D (5%)	-	0.8–1.6	-	MABA	Highly active	[29]
*Micromeria barbata* (Lebanon)	unspecified	-	1/1000 **	1/250 ** (MDR)	Broth microdilution	***	[30]
*Ocimum sanctum*, holy basil, tulsi (India)	unspecified	-	2.931	5.862 (S) 1.465–5.862 (MDR)	BD BACTEC MGIT	Highly active	[31]
*Plectranthus amboinicus* syn. *Coleus amboinicus*, Mexican mint (Brazil)	thymol (61.5%), β-pinene (11.2%), γ-terpinene (10.2%), caryophyllene (5.3%)	-	351.6 ± 39.06	-	REMA	Moderately active	[22]
*Premna odorata* (Egypt)	*trans*-caryophyllene (24.488–29.403%), β-phellandrene (11.701–22.930%), germacrene D (8.496–9.389%), Δ-cadinene (4.289–7.429%), α-humulene (3.529–7.016%), calamene (3.152–5.103%)	100 μL/mL ****	-	-	MMA-ELISA	Good	[32]
*Salvia aratocensis* (Columbia)	epi-α-cadinol (20.1%), 1,10-di-epi-cubenol (14.2%), γ-cadinene (9.3%)	-	62.5	49.6–99.2 (MDR)	Colorimetric macrodilution method	Highly active	[33]
*Salvia aucheri* subsp. *aucheri*, Turkish tea sage (Turkey)	1,8-cineole (39.2%), camphor (20.7%)	196	-	-	MGIT fluorometric method	Good	[34]
*Salvia tomentosa*, balsamic sage (Turkey)	α-pinene (25.1%), camphor (14.9%), borneol (13.2%), 1,8-cineole (7%)	196	-	-	MGIT fluorometric method	Good	[34]
*Satureja khuzestanica* (Iran)	unspecified	-	156	156 (MDR)	Broth microdilution	Good	[35]
*Satureja rechingeri* (Iran)	unspecified	-	78	156 (MDR)	Broth microdilution	Highly active to Good	[35]
*Tetradenia riparia*, ginger bush (Brazil)	α-cadinol (13.81%), 14-hydroxy-9-epi-caryophyllene (12.70%), 6,7-dehydroroyleanone (12.51%)	-	62.5	62.5 (S) 31.2–62.5 (MDR)	REMA	Highly active	[24]
*Thymus vulgaris*, common thyme (Iran)	unspecified	-	0.5–40	-	Unspecified	Highly active	[36]
*Zataria multiflora*, Shirazi thyme (Iran)	unspecified	-	78	78 (MDR)	Broth microdilution	Highly active	[35]

* for EOs/plant extracts: MIC < 100 μg/mL, highly active; MIC = 100–200 μg/mL, good activity; MIC = 200–500 μg/mL, moderately active; MIC = 1000–2000 μg/mL, poorly active; MIC > 2000 μg/mL, inactive [22,23]; ** higher essential oils dilution conferring a complete growth inhibition against the studied strains [30]; *** the presentation of antimycobacterial potency in that study does not allow an accurate evaluation of potential within the framework of the utilized MIC scale; **** at this concentration, EO determines a significant reduction in MTB antigen level comparing to the positive control group with measured values > 1.5 µg/mL MTB antigen [32]. EO, essential oil; MABA, Microplate Alamar Blue assay; MDR, multi-drug resistant; MIC, minimum inhibitory concentration; MGIT, Mycobacteria Growth Indicator Tubes; MMA-ELISA, Medipro *Mycobacterium tuberculosis* antigen-enzyme-linked immunosorbent assay; MTT, (3-(4,5-dimethylthiazol-2-yl)-2,5-diphenyl-2H-tetrazolium bromide; REMA, Resazurin Microtiter Assay; S, susceptible.

**Table 2:** Antimycobacterial activity of Lamiaceae volatile terpenes.

Compound	Lamiaceae Plant Sources	MIC (μg/mL)			Experimental	Potential *	Ref.
		H37Ra	H37Rv	Clinical Isolates			
Carvacrol	*Origanum vulgare*, *Origanum dictamnus*, *Satureja hortensis*, *Satureja montana*, *Thymus vulgaris*, *Thymbra capitata*	64	-	-	Broth microdilution	Good	[37]
		-	2.02 ± 0.88	-	MABA	Highly active	[38]
		-	76	19–76 (MDR)	REMA	Weakly active to good	[39]
		-	6.249	-	MABA	Highly active	[40]
(β)(*trans*)- Caryophyllene	*Ocimum basilicum*, *Origanum vulgare*, *Melissa officinalis*, *Lavandula angustifolia*, *Rosmarinus officinalis*, *Salvia* sp.	-	100	-	MABA	Weakly active	[38]
Caryophyllene oxide	*Hyptis stricta*, *Melissa officinalis*, *Mentha piperita*, *Mentha longifolia*, *Salvia* sp.	-	200	100 (MDR)	MABA	Weakly active	[41]
(β)-Citronellol	*Dracocephalum moldavica*, *Melissa officinalis*, *Plectranthus neochilus*	-	6.25	-	MABA	Highly active	[38]
*p*-Cymene	*Thymus vulgaris*, *Origanum vulgare*, *Ocimum gratissimum*	-	91.66 ± 14.43	-	MABA	Weakly active	[38]
6,7-Dehydroroyleanone	*Tetradenia riparia*	-	31.2	31.2	REMA	Good	[24]
			>25	≤12.5	Colorimetric MTT/ sulforhodamine B dye	Good to highly active	[42]
Estragole	*Ocimum basilicum*, *Ocimum* sp., *Agastache rugosa*	-	20.83 ± 7.22	-	MABA	Good	[38]
Eugenol	*Ocimum sanctum*, *Ocimum gratissimum*, *Agastache rugosa*, *Agastache foeniculum*	-	25	-	MABA	Good	[38]
		-	2.67 × 10^3^	2.67–10.68 × 10^3^ (S) 2.67–5.34 × 10^3^ (MDR)	BD BACTEC MGIT	Weakly active	[43]
		-	12.497	-	MABA	Good	[40]
Limonene	*Agastache urticifolia*, *Mentha* sp., *Dracocephalum* sp., *Rosmarinus* sp.	32	-	-	Broth microdilution	Good	[44]
Linalool	*Lavandula officinalis*, *Ocimum basilicum*, *Salvia sclarea*	-	33.33 ± 14.43	-	MABA	Good	[38]
Menthol	*Mentha piperita*, *Mentha arvensis*	-	41.66 ± 14.43	-	MABA	Good	[38]
		-	24.987	-	MABA	Good	[40]
Menthol-based ciprofloxacin derivatives	-	-	0.5–1	8–16 (MDR)	Broth microdilution MABA	Highly active	[27]
α-Pinene	*Rosmarinus officinalis*, *Salvia officinalis*, *Mentha piperita*, *Hyptis* sp.	128	-	-	Broth microdilution	Weakly active	[37]
		-	-	≥512	Serial dilution	Weakly active	[45]
Thymol	*Thymus vulgaris*, *Thymus* sp., *Origanum* sp., *Satureja hortensis*, *Satureja montana*	-	0.78 ± 0.01	-	MABA	Highly active	[38]
		-	50	-	MABA	Good	[40]
Thymol and carvacrol	*Thymus* sp., *Origanum* sp.	-	-	100	MABA	Weakly active	[46]
Thymol-based ciprofloxacin derivatives	-	-	0.5	16–32 (MDR)	Broth microdilution MABA	Highly active to good	[27]

* for pure compounds: MIC < 10 μg/mL, highly active; MIC = 11–64 μg/mL, good activity; MIC > 64 μg/mL, weakly active [24,25,47]; MABA, Microplate Alamar Blue assay; MDR, multi-drug resistant; MIC, minimum inhibitory concentration; MGIT, Mycobacteria Growth Indicator Tubes; MTT, (3-(4,5-dimethylthiazol-2-yl)-2,5-diphenyl-2H-tetrazolium bromide; REMA, Resazurin Microtiter Assay; S, susceptible.

**Table 3:** Inhibition of mycobacterial enzymes by terpenes present in Lamiaceae EOs.

Enzyme	Function	Compound	IC_50_/Activity	Ref.
Chorismate mutase (CM)	Aromatic amino acid biosynthesis; Modulation of cytokine response; Bacterial virulence	Carvacrol	1.06 ± 0.4 μM	[40,52]
		Thymol	28.4 ± 2.4 μM	
		Menthol	26.38 ± 2.1 μM	
		Eugenol	28.58 ± 1.7 μM	
Enoyl-acyl carrier protein reductase (InhA)	Biosynthesis of fatty acids and lipids of the cell envelope	Caryophyllene oxide, δ-cadinene	Higher affinities than triclosan, a standard inhibitor	[48]
		α-Terpinyl acetate	Comparable affinity for enzyme inhibition as isoniazid	[53]
Mycobacterial proteasomal ATP-ase (Mpa)	Degradation of damaged/ unnecessary proteins; Bacterial survival during hostile conditions	Germacrene D, τ-cadinol, δ-cadinene	Higher affinities than oxathiazol-2-one, a standard inhibitor	[48]
Decaprenylphosphoryl-d-ribose oxidase (DprE1)	Cell wall synthesis	R-Limonene	Down-regulation of gene expression; Decrease gene transcript levels	[44]
clgR	Preservation of cell membrane integrity and tolerance to surface stress; Macrophage infection; Stress-sensing marker	R-limonene	Increases gene expression similar to the positive control (sodium dodecyl sulfate)	[44]
EmbA-EmbB-AcpM2 arabinosyltransferase complex (EmbA-EmbB)	Cell wall synthesis Biological target for ethambutol	Caryophyllene oxide, *trans*-caryophyllene	Inhibition of the enzyme and its MDR-mutant variant	[54]

IC_50_, half-maximal inhibitory concentration.

**Table 4:** Combinatorial effects of terpenes from Lamiaceae EOs and antimycobacterial drugs.

Compound	Antimycobacterial Drug	Interaction	FICI	*M. tuberculosis*	Ref.
Carvacrol	Isoniazid	Additive	0.75	H37Rv	[39]
	Rifampicin	Synergism	0.25–0.40	MDR	
	Ethambutol	Additive	0.60	MDR	
Eugenol	Ethambutol	Additive	0.62	H37Rv	[78]
	Rifampicin	Synergism	0.25	H37Rv	
	Rifampicin	Synergism	0.132–0.5	MDR	
	Isoniazid	Synergism	0.26–0.43	MDR	
	Ethambutol	Synergism	0.31–0.5	MDR	
	Pyrazinamide	Synergism	0.31–0.37	MDR	
(R)-Limonene	Ethambutol	Synergism	0.30	H37Ra	[45]
	Rifampicin	Synergism	0.10	H37Ra	
(S)-Limonene	Ethambutol	Synergism	0.10	H37Ra	
	Isoniazid	Additive	0.60	H37Ra	
	Rifampicin	Synergism	0.10	H37Ra	
Myrcene	Ethambutol	Synergism	0.20	H37Ra	
	Rifampicin	Synergism	0.10	H37Ra	
α-Pinene	Rifampicin	Synergism	0.10	H37Ra	

FICI, fractional inhibitory concentration index. FICI ≤ 0.5 indicates synergy, 0.5 < FICI ≤ 1 (or <4 depending on framework) shows additive/indifferent effects, and FICI ≥ 4.0 defines antagonism [45,78].

## Data Availability

No new data were created or analyzed in this study. Data sharing is not applicable to this article.
